# Focusing in bioproduction science

**DOI:** 10.1186/1475-2859-4-10

**Published:** 2005-04-06

**Authors:** Antonio Villaverde

**Affiliations:** 1 Institut de Biotecnologia i de Biomedicina and Departament de Genètica i de Microbiologia, Universitat Autònoma de Barcelona, Bellaterra, 08193 Barcelona, Spain

## Abstract

As in other Biotechnological fields, the microbial production of recombinant proteins and other biomolecules can be approached from multiple angles through the help of diverse technologies of increasing complexity. To better reach all the specialized niches in bioproduction, Microbial Cell Factories is now inviting authors to prepare concise Reviews (eventually miniReviews), covering relevant areas that deserve specific and highly focused attention. By the publication of such contributions, the journal will promote the revision of new insights around the Cell Factory concept in a highly comprehensive way, in molecular, cellular and environmental contexts.

## Editorial

The spontaneous diversification of science is notably increasing the number of niches in which scientists specialize. Therefore, despite the multidisciplinary profile of current scientific approaches, the speedy enrichment of defined conceptual areas generates multiple "hot spots" with a particular interest. To this end, updating the scientific and technological record through the publication of Reviews, requires a detailed coverage of such fields, that while keeping a broad interest need to be of additional value for specialists.

Bioproduction science is not distinct from this universal stream. The growing number of host cell systems being explored as factories, the rising types of bioproducts, the continuous improvement of vectors and the gene expression setting-up, the increasing awareness of the host cell physiology and stress responses occurring under controlled production, and the implementation of new technologies for strain selection-improvement and for process control-monitoring are demanding more refined, topic-oriented attention.

Therefore, apart from the publication of broad standard Reviews, Microbial Cell Factories is adjusting its policy to encourage the publication of highly focused Reviews (eventually miniReviews), covering core aspects of the Cell Factory concept with self-contained interest. Authors are invited to submit proposals for such concise Reviews, that are to be prepared under the general journal Review style . Such contributions, whilst of general interest in the bioproduction landscape, will particularly fulfill the changing needs within highly specialized technical and scientific areas.

